# Phylogeographic analysis of the Bantu language expansion supports a rainforest route

**DOI:** 10.1073/pnas.2112853119

**Published:** 2022-08-01

**Authors:** Ezequiel Koile, Simon J. Greenhill, Damián E. Blasi, Remco Bouckaert, Russell D. Gray

**Affiliations:** ^a^Department of Linguistic and Cultural Evolution, Max Planck Institute for Evolutionary Anthropology, Leipzig 04103, Germany;; ^b^Linguistic Convergence Laboratory, National Research University Higher School of Economics, Moscow 105066, Russia;; ^c^School of Biological Sciences, University of Auckland, Auckland 1010, New Zealand;; ^d^Department of Human Evolutionary Biology, Peabody Museum, Harvard University, Cambridge, MA 02138;; ^e^Human Relations Area Files, Yale University, New Haven, CT 06520;; ^f^Centre for Computational Evolution, University of Auckland, Auckland 1142, New Zealand;; ^g^School of Psychology, University of Auckland, Auckland 1142, New Zealand

**Keywords:** Bantu expansion, phylogeography, linguistic geography, Central African rainforest

## Abstract

The Bantu expansion transformed the linguistic, economic, and cultural composition of sub-Saharan Africa. However, the exact dates and routes taken by the ancestors of the speakers of the more than 500 current Bantu languages remain uncertain. Here, we use the recently developed “break-away” geographical diffusion model, specially designed for modeling migrations, with “augmented” geographic information, to reconstruct the Bantu language family expansion. This Bayesian phylogeographic approach with augmented geographical data provides a powerful way of linking linguistic, archaeological, and genetic data to test hypotheses about large language family expansions. We compare four hypotheses: an early major split north of the rainforest; a migration through the Sangha River Interval corridor around 2,500 BP; a coastal migration around 4,000 BP; and a migration through the rainforest before the corridor opening, at 4,000 BP. Our results produce a topology and timeline for the Bantu language family, which supports the hypothesis of an expansion through Central African tropical forests at 4,420 BP (4,040 to 5,000 95% highest posterior density interval), well before the Sangha River Interval was open.

## Significance

Southern Africa has been shaped by the large-scale expansion of Bantu populations fueled by agriculture: Currently, 240 million people speak one of the more than 500 Bantu languages. However, the timing and geographic routes undergone by the Bantu populations remain largely unknown. We use cutting-edge phylogeographic techniques to show that Bantu populations migrated through the Central African tropical rainforest around 4,400 y ago. This adds to the growing evidence that agricultural expansions can successfully overcome ecological challenges as they unfold.

The Bantu expansion was a massive migration that reshaped the linguistic and cultural landscape of Africa. It led to the proliferation of Bantu-speaking populations throughout sub-Saharan Africa, and, today, more than 500 languages classified as “Bantu” are spoken by 240 million people across an area of 9 million square kilometers (1). This expansion has been associated with major economic and cultural changes across sub-Saharan Africa, including a more sedentary way of life, iron working, and crop cultivation (2, 3). Plants that are significant to subsistence across Africa today, such as pearl millet (*Pennisetum glaucum*), cowpea (*Vigna unguiculata*), and fonio (*Digitaria sp.*), have names of Bantu origin, suggesting that agricultural innovations fueled the expansion of this language family (4, 5). The origin or “homeland” of this process is generally believed to be near the border of Nigeria and Cameroon (6), and its time of origin is believed to be between 4,000 and 5,000 y BP (7, 8).

Despite our knowledge about this expansion, substantial uncertainty remains about the route and environmental conditions faced by early Bantu-speaking populations as they expanded from this point in a southward path across Africa. Robust linguistic and genetic evidence (1, 3, 9–11), as well as a general lack of preexisting hunter-gatherer populations documented archaeologically in much of the current Democratic Republic of the Congo (12), indicate that the current distribution of the Bantu populations is mainly due to a population expansion, as opposed to cultural diffusion, whereby the languages—rather than the speakers—spread through their progressive adoption by the local hunter-gatherer groups. The fundamental challenge posed by this picture is the presence of the massive Central African tropical rainforest which, at the time of the Bantu expansion, covered the region between the Atlantic coast and the African Great Lakes. Tropical rainforests, both in Africa (3) and elsewhere (13, 14), have been considered a barrier to the expansion of agricultural groups. Poor soils and difficulty of navigation have been seen as particularly problematic for the expansion of “dry” crops such as pearl millet and fonio (3), which are the staple source of nutrition of Bantu peoples. In order to account for the hurdle imposed by the rainforest in the history of the Bantu expansion, three main hypotheses have been proposed in the literature so far, each receiving different degrees of support from genetics, linguistics, and archaeology.

## Early-Split Hypothesis

**Fig. 1.**
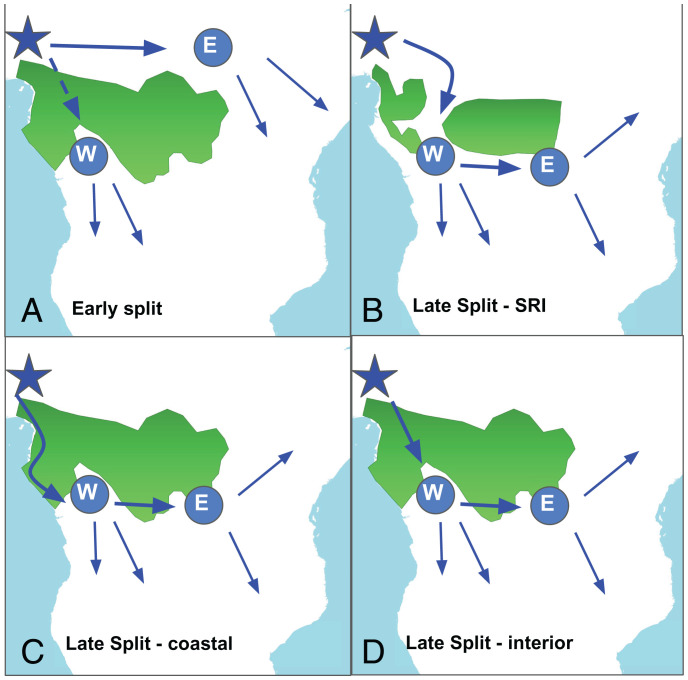
Hypotheses of the early migration of Bantu peoples. *A* shows the early-split hypothesis, while *B*–*D* show the different versions of the late-split hypothesis: (*B*) through the Sangha River Interval, (*C*) via a coastal route, and (*D*) through the interior of the rainforest. In all cases, the star stands for the homeland, while E and W stand for the East and West Bantu branches, respectively. The approximate rainforest extensions for 5,000 BP (*A*, *C*, and *D*) and for 2,500 BP (*B*) are based on refs. 29 and 30.

Bantu languages have traditionally been grouped into two major branches, Eastern and Western, on the basis of extensive linguistic scholarship, which has served as a main starting point from which to investigate the history of the Bantu expansion (15–20). Eastern Bantu languages cover the region east of the African Great Lakes, from the region around Lake Victoria in the north to modern-day South Africa in the south, whereas Western Bantu languages range from the Guinea Gulf in the north to the north of modern Namibia in the south. The early-split hypothesis (Fig. 1*A*) proposes that these two branches had split already during the peopling of the homeland in West Africa. According to this hypothesis, only West Bantu speakers entered the rainforest, while the East Bantu branch avoided it by moving eastward toward the African Great Lakes region, first, and heading south afterward. East–west migrations of agricultural populations are, in general, more common than north–south migrations, since the former face a more gradual variation in climate and habitat, which facilitates the spread of crops (21).

## Late-Split Hypothesis through the Sangha River Interval

By contrast, several linguistic and genetic studies suggest that East Bantu is one independent linguistic branch that split from the main West Bantu branch, after the rainforest was traversed (22–25). It has been hypothesized (26, 27) that this traversal was possible as a result of the “Late Holocene Rainforest Crisis” (28), a sudden shrinkage of the African forests which occurred between 3,000 and 2,500 y BP. During this event, primary forest trees were greatly reduced, and a major expansion of savannas took place (29, 30). In this context, a 400-km-wide corridor, known as the Sangha River Interval, opened, connecting the north and the south ends of the rainforest zone, enabling dry crops to be effectively transferred and grown through the rainforest, thus allowing for continuity in the Bantu agriculturalist practices. According to the paleoclimatic dating of these events, this hypothesis implies that the Eastern branch emerged only *ca.* 2,000 BP, after the corridor was completely open and posterior southward migration of the Bantu populations occurred (Fig. 1*B*).

## Late-Split Hypothesis through a Coastal Route

Although the previous hypothesis would account better for the general topology of the Bantu linguistic tree (the East branch emerging out of the West branch, instead of an initial early split), it fails to explain the time depth of the existing archaeological evidence. East Bantu settlements dated to 3,000 y BP have been found more than 1,500 km beyond the Sangha River Interval, suggesting a much earlier migration through the rainforest (31). As a consequence, a third hypothesis has been proposed, consistent both with the late-split topology observed in the linguistic reconstructions and with an earlier divergence time for the Eastern branch. This envisions a migration through a coastal route that surrounded the rainforest, skirting modern-day Gabon (32–34) (Fig. 1*C*). According to this hypothesis, coastal plains or drier forests—which are found near the Atlantic Ocean—could have provided useful pathways for the expansion of farming. This hypothesis allows for an earlier divergence time for the two main Bantu branches, *ca.* 4,000 BP.

## Late-Split Hypothesis through the Rainforest Interior

Previous geographic reconstructions of the Bantu expansion (3, 35, 36) consistently find a migration route in the interior of the rainforest, far from the proposed coastal route. Although these studies tend to interpret this result as supporting the late split through the Sangha River Interval hypothesis (this is not the case for ref. 36, which suggests rivers and valleys as natural corridors of migration), this interpretation yields an inconsistent dating of the migration events (3, 31). In dated reconstructions (3), the age of the Eastern branch is 2,500 BP. We would expect the Eastern branch to postdate the opening of the savanna corridor which only fully opened around 2,500 BP, therefore it is hard to reconcile this age with an expansion through the corridor. Alternatively, we could take the predictions of these models at face value and consider a migration through the interior of the rainforest, before the opening of the Sangha River Interval, which has been disregarded, as implausible, until now. Recent developments in the study of human–rainforest interactions (in Central Africa and elsewhere) have triggered a profound rethinking of the likelihood of an agricultural expansion through the Central African rainforest. To start with, tropical forests in Central Africa are incredibly diverse, and not homogeneously characterized by dense evergreen rainforest—which cannot be easily traversed. Instead, more-open forest types near river courses (which are plentiful in the Congo Basin) may have offered significant pathways of movement (37). Furthermore, while studies show that major tropical forest retreat did not occur until 3,000 BP to 2,000 BP, it is possible that drier forest types—which would have been appealing to cultivators—began to dominate significant portions of what would become the Sangha River Interval much earlier. In addition to this, growing evidence shows that human societies dramatically modified the Central African rainforest through slash-and-burn practices, creating a niche for agricultural lifestyles, with noticeable effects already by 3,000 BP (38, 39, cf. ref. 40).

Finally, the subsistence of the expanding agriculturalist groups might have been more complex than is often described, as proposed by Klieman (41) based on the theory of a “slow revolution” of farming in subequatorial Africa (42). According to this account of archaeological and linguistic data, Bantu settlers 1) left West Cameroon with a knowledge of agriculture centered on the cultivation of root crops and 2) used stone axes and digging sticks to prepare and plant fields, and 3) hunting and fishing were important sources of subsistence, probably learned from earlier neighboring populations. According to this reference, the acquisition of pottery allowed for demographic growth, and polished stone axes and hoes helped clear settlements in the forest. Migrations would have occurred along major rivers, several centuries before the full opening of the Sangha River Interval.

Putting all the strands of evidence together, we introduce a fourth hypothesis which would account for all the facts of the Bantu expansion: a late divergence between Eastern and Western Bantu branches after passing through the Central African rainforest well before the opening of the Sangha River Interval (Fig. 1*D*; see also ref. 41; cf. ref. 12).

In the present paper, we evaluate the plausibility of these four hypotheses (Fig. 1), using a state-of-the-art Bayesian phylogeographic approach applied to large-scale vocabulary data, and historical, archaeological, and paleoclimatic evidence.

## Phylogeographic Approaches to Language Expansions

Given the demic nature of the Bantu language expansion, phylogenetic inference (43) and, more concretely, phylogeographic methods have been a fundamental tool in reconstructing its geographic route and origins (44). These models usually consider a random walk through continuous space along the branches of a tree (45–47) which, combined with the cognate-coded linguistic data, allows joint reconstruction of linguistic and geographical history. While the underlying assumptions might not hold for a number of attested language histories (48, 49), this methodology has proven fruitful in testing different migration and expansion hypotheses across language families and regions of the world (50), including those relating to Austronesian (51), Indo-European (45, 52, 53), Dravidian (54), Pama-Nyungan (55), Semitic (56), Sino-Tibetan (57, 58), and Tungusic languages (59). Similarly, phylogenetic studies of the Bantu languages and their populations have successfully enhanced our understanding of the dynamics and the dating of the expansion (1, 3, 10, 11, 35, 36).

However, the modeling assumptions underlying these methods could give rise to biased inferences, either because they might be at odds with what is known about the dynamics of human groups, or because of limitations in the modeling of space, or because they are derived from a partial analysis of the populations under study.

### Challenge 1: Dynamics of Human Migrations.

Standard phylogeographic models approximate the spread and diversification of languages with a simple diffusion model. In particular, most standard models assume that, after a language splits into a number of descendants, the descendants spread spatially with the same speed in random directions (3, 45, 47, 60, 61). However, this is not realistic, as many well-attested migratory histories reveal starkly different dynamics. For instance, Austronesian languages spread through the Pacific in a sequence of expansion pulses and settlement pauses (51). One of the major consequences of assuming a simplified, equal-rate, dynamics is that it tends to allocate the putative homeland of a group of languages somewhere close to their geographic centroid. This is clearly not the case for most well-studied language families: The Austronesian (51), Sino-Tibetan (57, 58), and Uto-Aztecan (62) all developed from the periphery of their present-day geographic distributions. This is also a concern in the case of the Bantu expansion, where robust scholarship has established the border between Cameroon and Nigeria as its homeland (6–8).

### Challenge 2: Spherical Geography.

Large linguistic families such as Bantu, Pama-Nyungan, Austronesian, and Uto-Aztecan cover a large latitudinal range. If latitude and longitude are treated as coordinates on a plane, this latitudinal extension generates a distortion in distances, because the actual geometry of the world’s area is better approximated by a sphere (55). While this could be ameliorated through specific coordinate transformations, the bias would persist at the extremes of the range of spatial extension, thus biasing inferences about the spatial process (45).

### Challenge 3: Geographic Sampling Bias.

Data availability differs substantially across languages and language groups, and, in general, 35 to 42% of the languages of the world remain to be described in detail (63). This problem is particularly critical for phylogeographic methods when the distribution of data availability is spatially structured. It has been shown that geographic sampling biases can lead to erroneous inferences in root location, migration rates, time depths and, in consequence, the emerging history of the groups under study (64, 65). In particular, the coverage of our sample on Bantu languages is skewed, being higher in regions such as the northeast of the Bantu-speaking region, while it is lower in others such as the southwest (*SI Appendix*, Fig. S1).

### Challenge 4: Multiple Waves of Migration.

It is often the case that several waves of population colonize a region, as has been proven for Southeast Asia (66, 67) and the Americas (68, 69). In particular, it could be that multiple population waves originating from the same homeland expand over similar territories. This has been argued to be the case during the Bantu expansion, based on archaeological evidence (70). Phylogeographic methods rely on nonlinguistic evidence (e.g., archaeological sites) for calibrating the dates of known events along the tree. However, in the case of a migration in multiple waves, some of these calibrated events might correspond to populations (and languages) that are different from the ones that will end up diversifying into the current languages we aim to model.

### A Robust and Realistic Model for the Bantu Expansion.

In this work, we analyze a large dataset of cognate-coded basic vocabulary from 419 Bantu and related Bantoid languages, and implement a model-based approach for building its phylogeography. We address the first and second challenges mentioned above by using a “break-away” or “founder-event dispersal” model (55). Under this model, population splits lead to one of the subpopulations staying in place and the other one diffusing away. This adequately captures the dynamics that take place when founder populations migrate to colonize a new territory (55). In addition, this model calculates the diffusion on a spherical surface representing the globe (rather than on a plane), therefore minimizing the distortion due to the large latitudinal range covered by the Bantu family (46).

We address the third challenge by tailoring a method used by geneticists when dealing with a similar issue. Despite the skewed data distribution for Bantu languages, we do have access to the approximate geographic coordinates of languages for which no cognate data are available, as well as historical linguistic judgments in relation to the affiliation of such languages (i.e., where they belong in the linguistic tree proposed through the classic comparative method) (71). With this information, we can produce multiple imputations of the unobserved languages by placing them in their putative clades, thus yielding a full tree of Bantu languages. This technique, known as “sequence-free” sampling, has proven to be very successful in alleviating the geographic sampling bias in genetics, although it has yet to be applied to linguistic data (65, 72, 73).

Finally, we want to make our reconstruction consistent with the possibility of two waves of Bantu populations migrating southward, as has been recently proposed (70). For this, we eliminate one calibration point, corresponding to East Bantu archaeological sites south of the rainforest, at 2,500 y BP (74). Keeping this calibration here would imply not only that current East Bantu speakers are direct descendants of the populations responsible for this archaeological site but also that the crossing of the rainforest was finished by this time, biasing the comparison of our hypotheses (*Materials and Methods* and *SI Appendix*, Fig. S6).

## Results

Here we present a Bayesian phylogeographic evaluation of the four hypotheses introduced in the previous sections. In summary, our analysis pipeline consists of three parts.

• Part 1: We produce a posterior distribution of dated linguistic phylogenetic trees, from cognate data taken from basic vocabulary.
• Part 2: Based on these trees and the geographic location of the Bantu and Bantoid languages, we produce estimates of the spatial spread of the Bantu expansion through their history.
• Part 3: We enrich our lexical database with sequence-free samples, leading to precise estimations of the biases introduced by geographical sampling.

### Part 1: Dated Phylogenetic Tree of Bantu Languages.

We deployed a Bayesian phylogenetic analysis for 419 Bantu and Bantoid languages’ lexical data (3, 75) (see *Materials and Methods*). We implemented our inference in the software BEAST 2, choosing the best site model and clock specification as resulting from a model selection approach (see *Materials and Methods*).

The resulting maximum clade credibility tree is shown in *SI Appendix*, Fig. S2, where the languages are grouped into 24 clades for display purposes (see also Dataset SS1 for the full detailed tree). The origin of the Bantoid languages is dated to 4,940 BP (95% higher posterior density interval [HPD] is 4,500 BP to 5,400 BP; root in *SI Appendix*, Fig. S2). The second oldest node, representing the split between narrow Bantu and the northwestern branch Mbam–Bubi (node 1), has a median age of 4,140 BP (95% HPD 3,950 BP to 4,380 BP). It is noticeable that two main branches emerge from this node, one containing the northwestern languages which populate the region currently covered by the rainforest, and the remaining containing the languages south and east of it.

Most of our 24 clades shown here can be traced to those in the consensus tree of a previous publication (3), with minor differences (e.g., a few mixtures between clades 12 and 13, and the split of clades 2 and 7; *SI Appendix*, Fig. S8). The main difference resides in the relations between these clades (see *Discussion* for further detail). That study, as well as previous Bantu phylogenies (1, 35, 36), present a “backbone” from which languages gradually split into smaller groups. This contrasts with our large split at 3,560 BP (95% HPD 3,330 BP to 3,820 BP, node 2). Also, we infer that the Central-Western branch is nonmonophyletic, but is, instead, divided into the two large subbranches from node 2 (*SI Appendix*, Fig. S8).

### Part 2: Geographic Model.

We implement a combined analysis, including a linguistic as well as a geographic model, in order to find an explicit migration route consistent with the tree topology obtained in the previous section. We use the break-away geographic model (55) implemented in BEAST 2 (76) as described in *Materials and Methods*. Notice that, in all cases, we describe an expansion of a single group of peoples. A recent study based on archaeological evidence (70), however, challenges this concept, arguing that a massive population collapse took place between 1,600 and 1,400 BP, and new waves of Bantu-speaking populations repopulated areas left empty by extinct earlier Bantu-speaking populations.

**Fig. 2.**
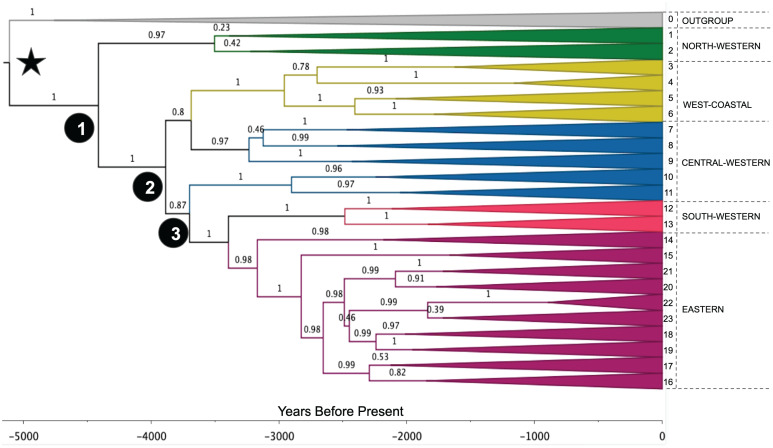
Maximum clade credibility tree, including the lexical and geographical information. The root is marked with a star, and main nodes (1 to 3 in black circles), as well as main clades (0 to 23), are numbered. Numbers on the branches represent the posterior support for their nodes. Notice the split in node 2, that generates a topology different from previous classifications, for example, making the Central-Western group nonmonophyletic (3, 35). Also, the West-Coastal group includes languages previously classified as North-Western (clades 3 and 4, corresponding to languages B10–B30), and the South-Western branch is monophyletic.

We obtain a posterior distribution of trees, whose maximum clade credibility tree is shown in Fig. 2. A more detailed tree showing the languages included in each clade can be found in *SI Appendix*, Fig. S3, and the full tree is shown in Dataset SS2. The median age of root is estimated to be 5,110 BP (95% HPD 4,640 BP to 5,770 BP), while the split between narrow Bantu and the northwestern branch Mbam–Bubi dates to 4,420 BP (95% HPD 4,040 BP to 5,000 BP).

Differences between the trees built solely on lexical material (previous subsection) and the combined lexical + geographical tree (this subsection) are relatively minor (compare *SI Appendix*, Fig. S8, *Left* and *Center*). The most important difference is the location of the language Sakata (C34), assigned to clade 5 (Njebe–Mbete–Teke) in the lexical tree, and reclassified with the combined model into clade 9 (Kela–Ntomba). This is consistent with the expert judgements reflected in Glottolog (71). *SI Appendix*, Fig. S4 shows the migration paths according to this reconstruction, and *SI Appendix*, Fig. S5 shows heatmaps with the posterior distribution for the locations of each relevant node.

### Part 3: Augmented Geographic Model.

**Fig. 3.**
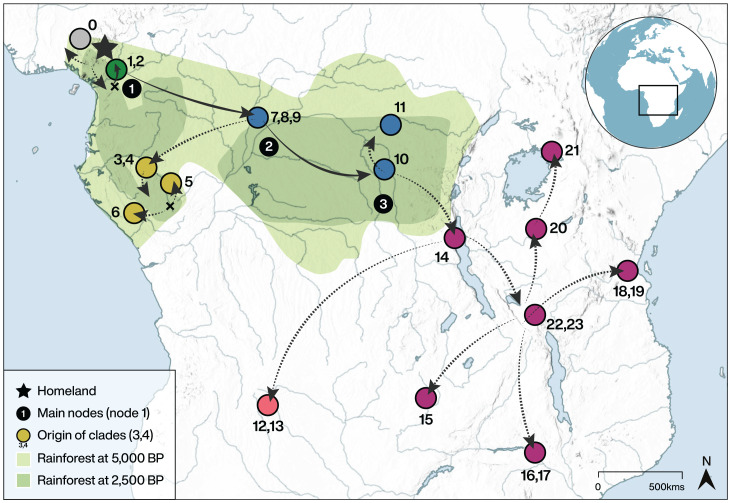
Bantu migrations reconstructed by using the break-away model in the augmented phylogeographic tree in Fig. 2. The homeland is marked with a star, and main nodes (1 to 3), as well as main clades (0 to 23), are numbered, following the notation and color coding in Fig. 2. Each colored circle represents the median value of the posterior distribution for the origin of the respective clade (see Fig. 4 for greater detail). Each black circle tags a node, whose exact location corresponds to the closest colored circle if it corresponds to the origin of a clade (nodes 2 and 3), or to the closest cross if not (as in node 1). The span of the rainforest at 5,000 BP and at 2,500 BP, according to refs. 29 and 30, is shown.

**Fig. 4.**
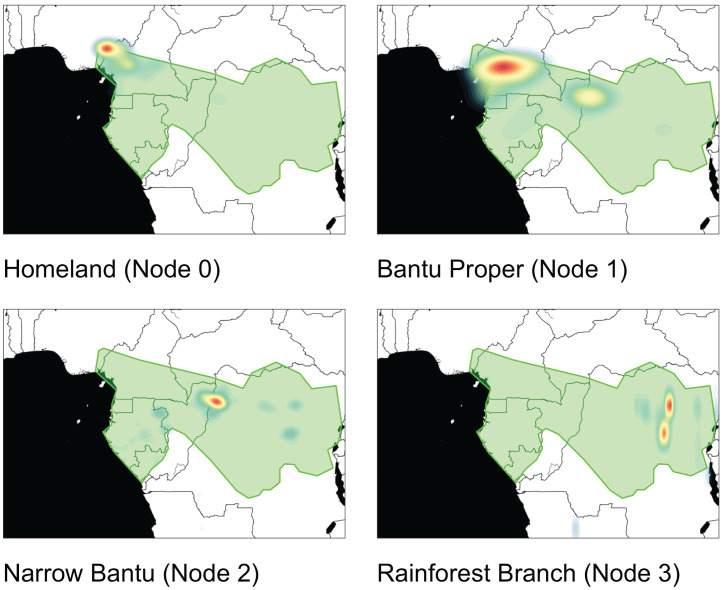
Heatmaps for the posterior distribution of the locations of the homeland and nodes 1 to 3, as indicated in Fig. 2, obtained with the augmented geographic model. These support the fourth hypothesis of a late split and migration through the interior.

We supplement the data of the 419 observed languages (403 Narrow Bantu, 9 Grassfields, 6 Jarawan, and 1 Tivoid) with “sequence-free” imputations in order to consider all 562 languages listed as Narrow Bantu (minus Jarawan) in Glottolog (71) (see *Materials and Methods*). The reconstructed migration routes are shown in Fig. 3, and detailed heatmaps with the posterior distribution for each relevant node are shown in Fig. 4. The locations of the root and the main nodes remain similar to those obtained in the previous analysis (compare with *SI Appendix*, Figs. S4 and S5). This allows us to conclude that the sampling bias is not driving our inferred history. The full augmented tree is shown in Dataset SS3.

In the following discussion, we will rely on the tree built in part 2 (Fig. 2), and the migrations’ map built in part 3 (Fig. 3). This selection allows for using the most precise family tree (built exclusively from available linguistic and geographic data) as well as the least biased migrations map (including the locations and broad groupings of the languages with missing lexical data). This is done in a consistent way, given the procedures followed to build the augmented tree (see *Augmented phylogeography*).

## Discussion

### Our Findings.

First of all, our analyses consistently show the region of the Guinea Gulf (around the border between current Nigeria and Cameroon) to be the homeland of the Bantu expansion (Node 0 in Fig. 4). This is aligned with a host of archaeological and linguistic evidence, as well as with previous phylogeographic reconstructions (1, 3, 6, 35, 36). The inception of the Bantu expansion from this homeland is inferred to be at 5,110 BP (95% HPD 4,640 BP to 5,770 BP), again in agreement with previous ethnographic, linguistic, and genetic scholarship (3, 7, 8, 77, 78).

Our analyses, however, reveal substantial differences in the topology of the inferred tree. Beyond minor disagreements in low-level groupings (see *Materials and Methods*), three major discrepancies were found. First, other inferred Bantu trees contain a central “backbone” from which languages gradually split into small groups (1, 3, 35, 36). Instead, we find those clades result from a large and relatively fast diversification event at 3,890 BP (95% HPD 3,590 BP to 4,370 BP; node 2 in Fig. 2). Second, our results show that the Central-Western branch of the Bantu tree (which encompasses 15% of all Bantu languages, including major ones such as Lingala) is not monophyletic as previously believed. Finally, we find that the B10–B30 languages, traditionally classified as North-Western, belong to the West-Coastal branch in our classification (clades 3 and 4 in Fig. 2). This is consistent with a recent suggestion based on phonological similarities (79).

Crucially, the dating and the geographic placement of the internal nodes of our tree strongly support the fourth hypothesis considered above: that Bantu peoples did travel through the Central African rainforest during their expansion. To start with, our analysis is decidedly consistent with a late split. The East Bantu branch splits from the rest of the tree only around 3,150 BP (95% HPD 3,030 BP to 3,830 BP, clades 14 to 23 in Fig. 2)—or 2,630 BP (95% HPD 2,400 BP to 3,090 BP), if we consider only clades 16 to 23, the proposed calibration point at 2,500 BP by ref. 3 from ref. 74)—which stands in stark contrast from what would be expected under the early-split hypothesis (*ca.* 4,000 BP to 5,000 BP). We then evaluated the geographic localization of the major split dividing languages within and far south and east from the rainforest. More concretely, we evaluate whether the Bantu expansion traveled through the rainforest directly or whether it adopted a coastal route instead, by comparing the relative concentration of the posterior distribution of the node in each of those regions (Node 2 in Fig. 4; see *Materials and Methods* for details). When comparing an interior vs. a coastal route, we obtain a Bayes Factor BF(interior/coastal) = 25.4 or 31.7, depending on the exact definition of the regions, thus lending strong support to the interior pathway (*SI Appendix*, Fig. S9).

Finally, in order to distinguish the last two hypotheses (expansion through the Sangha River Interval vs. through the rainforest), we compare the dates of the first internal split of Narrow Bantu languages (node 2). The reconstructed age of crossing the rainforest is 3,890 BP (95% HPD 3,590 BP to 4,370 BP). Although there is evidence of climate changes generating intercalary savannas as early as 4,000 BP in the region that would become the Sangha River Interval (see *Late-Split Hypothesis through the Rainforest Interior*, and, e.g., refs. 3 and 31), our result is significantly earlier than the complete opening of the Sangha River Interval (*ca.* 2,500 BP). Therefore, only the rainforest route hypothesis is supported by our analyses.

### The Rainforest Route Hypothesis.

Our combined phylogeographic model reveals an early and “interior” route of dispersal of the Bantu peoples through Central Africa. This implies that Bantu-speaking groups expanded through the Central African rainforest, consistent with Klieman’s (41) proposal.

Our findings shed light on the substantial number of archaeological sites along the Sangha River Interval dated around 2,400 BP, which attest to pearl millet farming [already domesticated by 4,000 BP in the Sahel region (80–83)] and significant iron working (28). This evidence has been interpreted as supporting the late-split Sangha River Interval hypothesis, which we have established not to be supported by our analyses. Moreover, forest recovery witnesses a decline in millet farming in the Late Iron Age (39, 84), and the dominance of oil palm–dominated farming that fits more comfortably within denser canopies (85–87).

It is also worth pointing out that existing methods for determining changes in tropical forest type—rather than their extent—remain coarse (28). Suggestions that pearl millet arrived in Central Africa as part of a multicropping package of oil palm, yams, and cowpea (88, 89) imply a potentially complex process of assimilation and mosaic land use rather than fully fledged “open” cultivation. Nevertheless, currently definitive archaeological evidence for pre-2,500 BP occupation of the interior of Central Africa is almost nonexistent, and this remains a hypothesis.

One puzzling aspect of our results that should be explored further is the divergence in the directions of migration after node 4, commencing around the position of clade 14 in Fig. 3. After the eastward migration inside the rainforest reached the African Great Lakes region, there followed a “backward” migration of the South-Western branch (clades 12 and 13), in a southwesterly direction. This about-turn in the direction of migration in the savanna after departing the rainforest is striking. Further studies, for example, implementing differential travel costs along rivers, could give us further insight into this issue.

## Conclusion

Our phylogeographic models recover the historical relationships between Bantu languages and, indirectly, populations with state-of-the-art precision. More importantly, these models allow us to address one of the most long-standing puzzles in the recent history of sub-Saharan Africa, as we find decisive support for an early Bantu migration through the interior of the Central African rainforest around 4,400 y BP. This appears to add to growing evidence that tropical rainforests must not necessarily present a barrier for the expansion of agricultural populations. The current lack of traces of intensive agricultural practices in the Central African rainforest route might suggest Bantu-speaking populations adopted a flexible subsistence mode. This could have been facilitated by local ecological changes triggered by humans (as those widely attested to in the “human niche construction” literature), although much remains to be learned in relation to the associated cultural adaptations. The potential consequences of our findings extend well beyond the Bantu-affiliated migrations, as they challenge the notion that agricultural expansions are entirely determined by assumed ecological conditions for the cultivation and exploitation of specific crops.

## Materials and Methods

### Data.

All data and code are available at the OSF repository https://osf.io/us3q5/?view_only=d54efdad94e3449cae4b533e877b3888.

### Lexical Data.

We used the lexical dataset from ref. 3.

These data were collected from dictionaries, theses, and fieldwork by the author of ref. 90, including 56 languages extracted from the Atlas Linguistique du GABon (75), selecting the 100 best-documented meanings from the 159 meanings in total, for a dataset of 424 languages. We excluded the five extinct languages in these data. The resulting dataset was converted into a binary-coded matrix with 3,859 cognate classes from 419 languages (403 Bantu and 16 Bantoid non-Bantu languages).

### Geographic Data.

Latitude and longitude data on the current location of the languages studied were taken from ref. 3, except for three languages for which there were no geographic data listed. For these, locations were taken from Glottolog (71, 91) in the cases where this was available (D313_Mbuttu_1919), and by replicating the location of their neighboring languages with the same Guthrie code, when data were not available (C401_Babati_1919 and C52_Soko_1919). We assume that these locations have not been substantially modified by the impact of recent events such as the Atlantic slave trade and colonialism.

### Calibration Points.

We considered the following calibration points drawn from ref. 3: 1) 5,000+ Bantoid, non-Bantu (92), 2) 4,000 to 5,000 Narrow Bantu (2, 29, 30, 93–96), 3) 3,000 to 3,500 Mbam–Bubi ancestor (97), and 4) 2,500 Eastern Bantu (74).

Following established best practice (98), we reimplemented these calibrations as log-normal distributions instead of uniform distributions as in the reference (*SI Appendix*, Fig. S6).

### Phylogeny.

#### Phylogenetic model.

We first evaluated the best-fitting model of cognate evolution for these data by comparing eight models combining three different parameters: 1) the model for the sites: Continuous Time Markov Chain (99) or Covarion (100), 2) adding or not gamma distributed site heterogeneity (*γ* = 1 or 4), and 3) a strict or relaxed clock for cognate evolution (101). We ran each analysis for 400,000,000 generations in BEAST 2 (76).

A model comparison was run with path sampling (102) in BEAST 2. The best-fitting model was the Continuous Time Markov Chain (CTMC), with gamma distributed site heterogeneity (*γ* = 4) and relaxed clock (*SI Appendix*, Table S1).

#### Phylogeographic model.

Phylogeographic models are based on a migration process among the nodes in a tree informed by the geographical locations of its tips. A simple geographical model based on random walks (46) assumes that, at a node in the tree, a population splits, and both resulting groups follow random walks along branches to the child locations. However, it is unlikely that both populations need to move: The settled location may have plenty of resources, which is why the population was there in the first place, and only one of the two populations needs to migrate. We use the phylogeographic “break-away” model (55), which models this behavior. It assumes populations split at internal nodes in the tree, and one population follows a random walk along a branch in the tree for the duration of the length of the branch to the child of the node. We found that the break-away model more accurately reconstructed the root location toward the northwest end of the sampled region.

The model used for the cognate data in the phylogeographic analysis is the one that fit the data best for the analysis without geography (see *Phylogenetic model*), namely, CTMC, *γ* = 4, relaxed clock. The main difference here is that we only used calibration points 1 and 2 as described in the previous subsection, and a third point, point 5, related to the first split among the Narrow Bantu languages. The latter was taken with a broad prior distribution including both 4,000 and 2,500 BP, in order to compare both hypotheses.

#### Augmented phylogeography.

If we plot the languages present in our database against all known Bantu languages (71), along with their geographic location, we can observe that the languages in our sample do not represent equally the total of the listed Bantu languages (*SI Appendix*, Fig. S1). We have a total of 419 varieties, composed of 403 Narrow Bantu and 16 other Southern Bantoid used as an outgroup (9 Grassfields, 6 Jarawan, and 1 Tivoid). If we compare them with the classification in Glottolog, these represent only 376 languages (361 Narrow Bantu and 15 other Southern Bantoid), since several of our varieties are counted there as dialects. Glottolog lists 556 Narrow Bantu languages, therefore leaving 195 languages for which we have no lexical data. However, we know two things about them: 1) the current geographical location of their speakers and 2) the phylogenetic grouping to which these languages belong, according to published sources (71). At the time of retrieving the data from Glottolog, Jarawan was not a subgroup of Narrow Bantu. Therefore, we do not include extra Jarawan languages (and the count of Narrow Bantu languages might slightly differ from the current one).

To avoid a possible bias in our results, we “augmented” the trees by adding these missing languages in their established phylogenetic positions. Firstly, we took the final posterior of lexical trees. This posterior distribution has 419 varieties corresponding to 376 languages according to Glottolog. For each tree in the posterior, we imputed the remaining 195 languages listed in Glottolog for which we have no lexical data (see *Results*), by randomly inserting them in their corresponding clade (*SI Appendix*, *Tree Imputation* and Fig. S1) with the help of the R package addTaxa (103, 104). We then ran the break-away model again, keeping the tree topology fixed.

#### Comparison with previous classifications.

The most complete phylogeny of Bantu languages to date is that by Grollemund et al. (3). Therefore, we start by comparing our classification with theirs (*SI Appendix*, Fig. S8, *Center* and *Right*). First, the overall topology is different: We observe a large split in early times (our node 2), while the authors of ref. 3 obtain a backbone topology (green circles in *SI Appendix*, Fig. S8). For instance, it takes four splits to reach the Eastern Bantu branch in our results, while it takes nine splits in ref. 3. Secondly, the Central-Western branch (blue clades, red circles in *SI Appendix*, Fig. S8, comprising most of languages in Guthrie zones C and D) is monophyletic in ref. 3, while it is divided into two subbranches by the split in node 2: most of languages C, on the one hand (clades 7, 8, and 9), and languages D plus C54 Turumba, C55 Lokele, and C52 Soko, on the other (clades 10 and 11).

Thirdly, we obtain a monophyletic West-Coastal (aka West-Western) branch, as expected, but it appears related to the North-Western B10–B30 branch (clades 3 and 4), which is not the case in other classifications (3) (yellow clades and blue circles in *SI Appendix*, Fig. S8). It has been shown that West-Coastal branch can be characterized by a common phonological innovation, distinguishing this group from most of other Bantu groups. This is the phonemic merger of the Proto-Bantu velar stops *g and *k due to the devoicing of *g when not preceded by a nasal. However, this same merger seems to have taken place in several languages of the B10–B30 branch as well (79). Finally, the South-Western branch is completely monophyletic in our case, in contrast to ref. 3, where it is divided into three groups, nested among themselves and with the Eastern Bantu branch (pink clades and circles in *SI Appendix*, Fig. S8).

Other phylogenies are those by Currie et al. (35) and Whiteley et al. (36). They also show a backbone topology, as in ref. 3. However, ref. 36 does not suggest a migration along the Sangha River Interval, but along rivers and river valleys, consistent with our results (i.e., independent of the opening time of the mentioned corridor), and with ref. 41.

Ehret (105, 106) makes a detailed reconstruction of Bantu migrations consistent with our results. It is based on combining phylogenetic outcomes with the evidence of lexical and phonological innovations such as refs. 18, 41, and 107–109.

As for the comparison with the recent article (110) on the West-Coastal Bantu languages, which included new detailed data for varieties in this region, we find our results mostly consistent with theirs. On the one hand, the Kikongo Language Cluster corresponds exactly with our clade 6. On the other hand, they especially focus on the B50–B80 languages, which belong in our clade 5. Both in our study and in ref. 3, clade 5 is monophyletic, and is further divided into a subgroup of the B80s, on the one hand, and a branch that further divides into B50s and B60–B70, on the other.* This differs from ref. 110 in which, although they found a vast monophyletic clade uniting all B50–B70 and some B80 languages, other B80 languages ended up in what they call Kikongo Language Cluster extended branch. For the homeland of West-Coastal Bantu, ref. 110 finds a homeland between Kamtsha and Kasai Rivers in the Democratic Republic of the Congo, slightly southeastward from our current results (*SI Appendix*, Fig. S7) and previous studies (10). However, although thorough in its linguistic study, ref. 110 models the B50–B70 homeland only on the basis of current (updated) locations of languages, not making use of an evolutionary model of the full Bantu family for this reconstruction, as we do in our current paper.

## Supplementary Material

Supplementary File

## Data Availability

Datasets, code, and figures have been deposited in a repository of the Open Science Framework (https://osf.io/us3q5/?view_only=d54efdad94e3449cae4b533e877b3888) (111). Previously published data were used for this work (3).

## Footnotes

^*^Clade 5 includes exactly the 32 varieties of B50–B80 plus C34 Sakata in our nongeographic model, as well as in ref. 3, and only the B50–B80 varieties in our geographic model.
