# Supplementary material for: Phylogeographic analysis of the Bantu language expansion supports a rainforest route
Source: Proc Natl Acad Sci U S A. 2022 Aug 1;119(32):e2112853119. doi: 10.1073/pnas.2112853119 (PMC9372543; doi:10.1073/pnas.2112853119)
Supplement: Supplementary File [file pnas.2112853119.sapp.pdf]

PNAS
www.pnas.org

Supplementary Information for

Phylogeographic analysis of the Bantu language expansion supports a rainforest route

Ezequiel Koile, Simon J. Greenhill, Damian E. Blasi, Remco Bouckaert, Russell D. Gray

Ezequiel Koile.
E-mail: ezequiel_koile@eva.mpg.de

This PDF file includes:

Supplementary text
Figs. S1 to S9 (not allowed for Brief Reports)
Tables S1 to S2 (not allowed for Brief Reports)
Legends for Dataset S1 to S5
SI References

Other supplementary materials for this manuscript include the following:

Datasets S1 to S5

Supporting Information Text

Calibrations

We considered the following calibration points drawn from Grollemund et al 2015 (references for each point in the main text, Materials and Methods):

- a) 5,000+ Bantoid, non-Bantu
- b) 4,000–5,000 Narrow Bantu
- c) 3,000–3,500 Mbam-Bubi ancestor
- d) 2,500 Eastern Bantu

We re-implemented these calibrations as log-normal distributions instead of uniform distributions as in the reference. Figure S6 below show the prior distributions for these calibrations.

Bayes factor calculation

We compare a migration through the coast and through the interior of the rainforest. We compare the prior and posterior probability for node 2 (Narrow Bantu) to be located inside each region. Figure S9 shows three different extensions for each region, and Table S2 shows the resulting Bayes factors considering each of these regions. We can see that in the maximal case, where both regions are adjacent, the Bayes factor is 31.7.

Tree imputation

Figure S1 shows the locations of the Bantu languages present in our sample as compared to the total Bantu languages. We have a total of 419 varieties, composed of 403 Narrow Bantu, and 16 other Southern Bantoid used as an outgroup (9 Grassfields, 6 Jarawan, and 1 Tivoid). All of these are shown in green in Figure S1. If we compare them with the classification in Glottolog, these represent only 376 languages (361 Narrow Bantu, and 15 other Southern Bantoid), since several of our varieties are counted there as dialects. Glottolog lists 556 Narrow Bantu languages, therefore leaving 195 languages for which we have no lexical data, but we know their locations and broad phylogenetic grouping according to published sources. These languages are shown in red in Figure S1. At the time of retrieving the data from Glottolog, Jarawan were not part of Narrow Bantu. Therefore we do not include extra Jarawan languages. Also, the count of 556 of Narrow Bantu languages might slightly differ from the current one, including Jarawan as Narrow Bantu.

For this imputation, we used the groupings shown in Glottolog, which base their classification off expert classifications, using the principles of historical linguistics, as explained in https://glottolog.org/glottolog/glottologinformation#principles. Requirements for including a language in a classification in Glottolog (different from “unattested” or “unclassifiable”) include: the availability of form-meaning pairs (i.e. not purely sociolinguistic data), and that these pairs are enough to distinguish between different classification proposals (approximately 50 items of basic vocabulary collected). Once these are satisfied, for it to be included in a family, it must show above chance similarities to other language(s), that can be explained by inheritance from a common ancestor. In order to be classified in a specific group beyond the family level, it is necessary to have sufficient comparison attested to determine its closest relative(s). For subgrouping, there must be shared innovations attested, or at least shared similarities in general, e.g., lexicostatistics, which may reflect borrowings and/or retentions.

To avoid a possible bias in our results, we “augmented” the trees by adding these missing languages in their established phylogenetic positions. For each tree in our posterior distribution, we imputed the remaining 195 languages listed in Glottolog for which we have no linguistic data, by randomly inserting them in their corresponding clade with the help of the R package addTaxa. We then ran the break-away model again keeping the tree topology fixed.

The procedure for this imputation was the following:

- First, we compared the distribution of the 376 languages that appear both in Glottolog and in our maximum clade credibility tree. We focused on the lowest level of classification in each tree in which the languages coincide. e.g.: Suppose that we have a group formed by languages A and B, and a different group formed by C and D in our tree: (A,B) and (C,D), while languages E* and F* are not in our lexical database.
- At this lowest level, if the group in Glottolog classification contains only languages from one group in our tree, plus languages not in our database, we took each tree in our posterior distribution, and randomly imputed the missing languages inside the corresponding group. In our example, Glottolog has a group formed by (A, B, F*), and another by (C,D,E*). Then, we added the new languages E* and F* in a random position inside the clades (A,B) and (C,D) respectively, augmenting the groups consistently.
- If these classifications were incompatible, and our groups were mixed with Glottolog’s to every level, we did not perform this task. In the example, Glottolog has one group (A, C, F*), and another separate group (B,D, E*). We ignore both E* and F* in these cases, avoiding to “break” the topology of our tree.

We therefore obtained a new posterior distribution of trees. This new distribution is clearly worse than the original one in terms of linguistic classification, for we have added noise from the random imputation of missing languages, but it is better in which it fixes the geographic sampling bias. The resulting geographic posterior distribution is more exact (for it takes into account the current locations of all known Bantu languages, even those for which we do not have linguistic data, in their approximate position in the tree), although possibly less precise (for it increases the noise). In addition, since the break-away model only allows ancient nodes to be placed in the geographic locations of existing nodes, this increase from 419 to 614 varieties (419+195) gives a larger space to infer nodes' locations.

The full list of languages, those with lexical data and those imputed, is in SI Dataset S1.

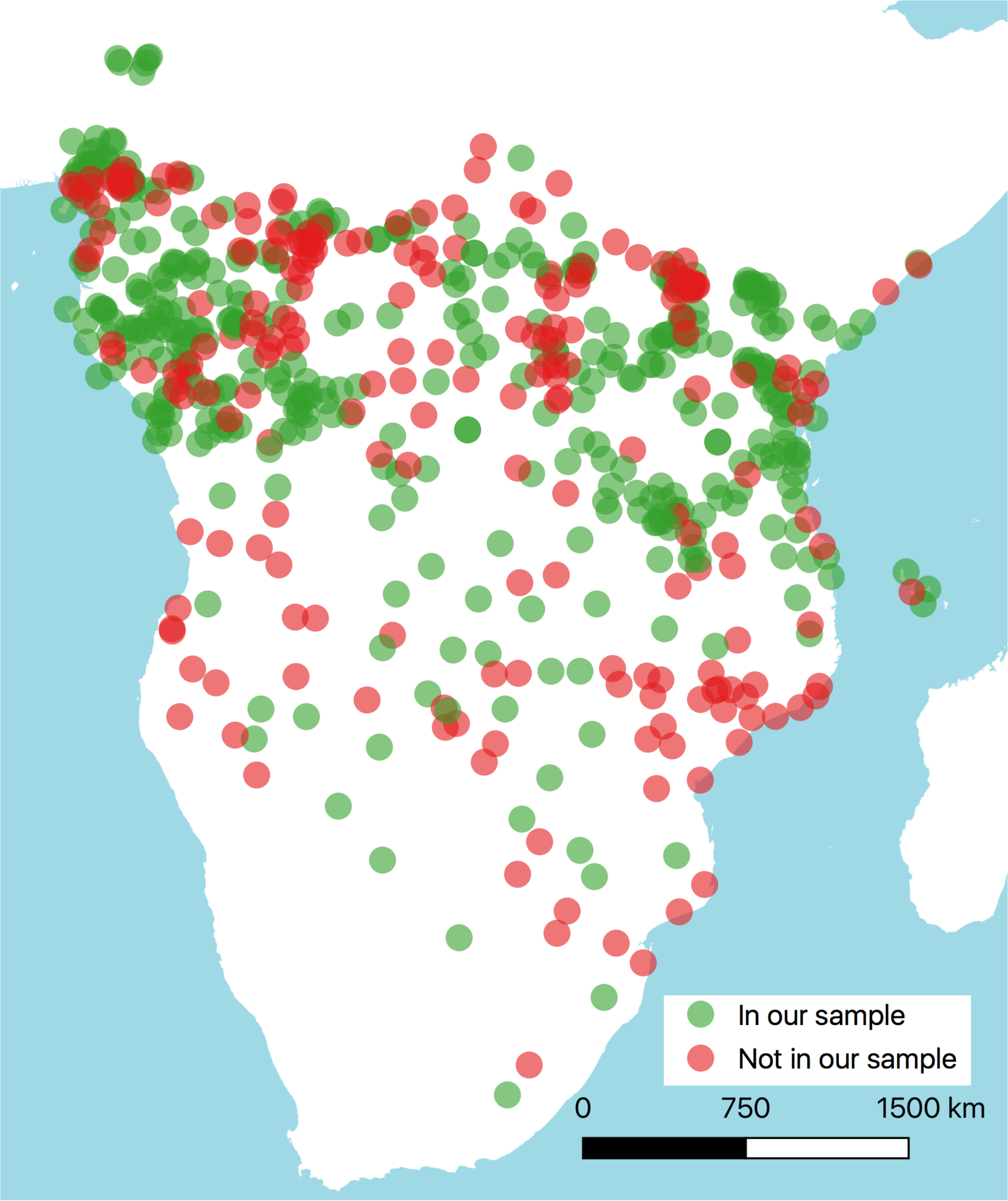

Fig. S1. Bantu varieties considered in our sample vs. those in Glottolog.

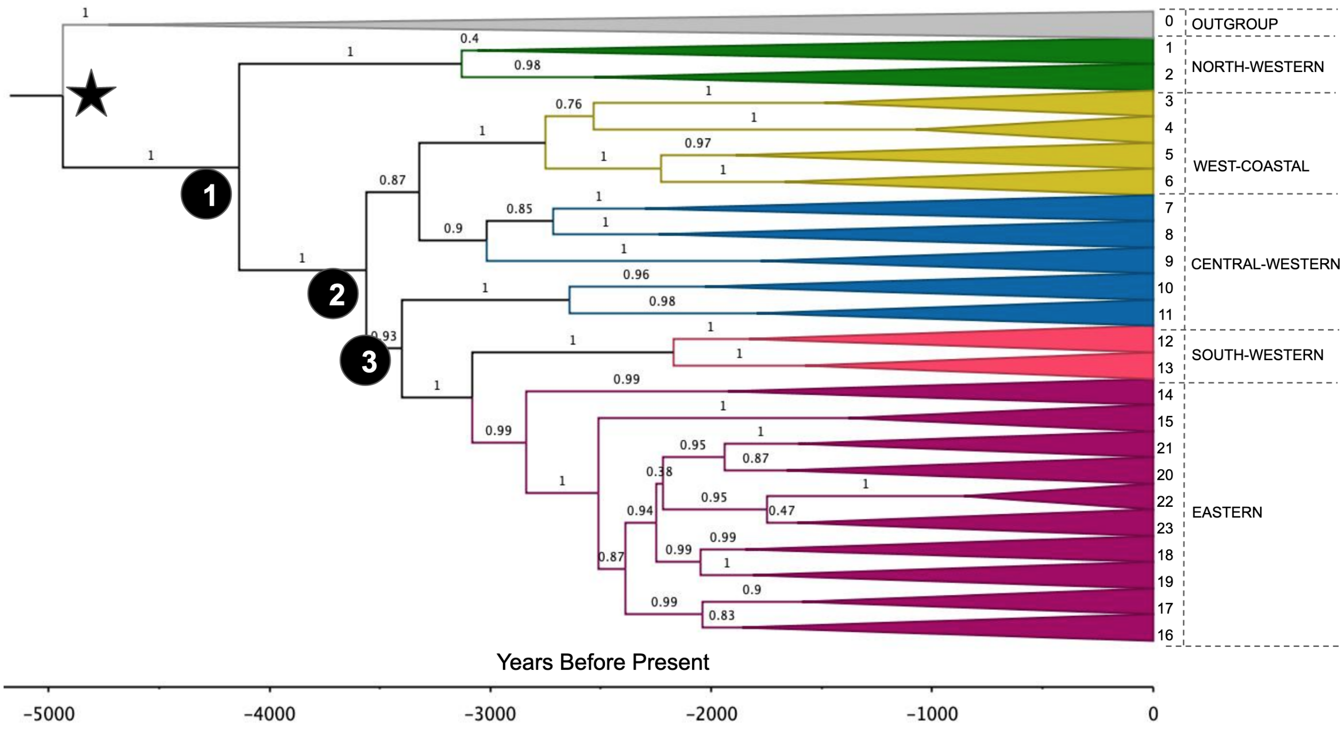

Fig. S2. Maximum clade credibility tree, including the lexical information only. The root is marked with a star, and main nodes are numbered (1-3), as well as main clades (0-23). Numbers on the branches represent the posterior support for their nodes. Notice the split in Node 2, that generates a topology different from previous classifications, e.g. making the Central-Western group non-monophyletic (1, 2). Also, the West-Coastal group includes languages previously classified as North-Western (Clades 3 and 4, corresponding to languages B10-B30), and the South-Western branch is monophyletic.

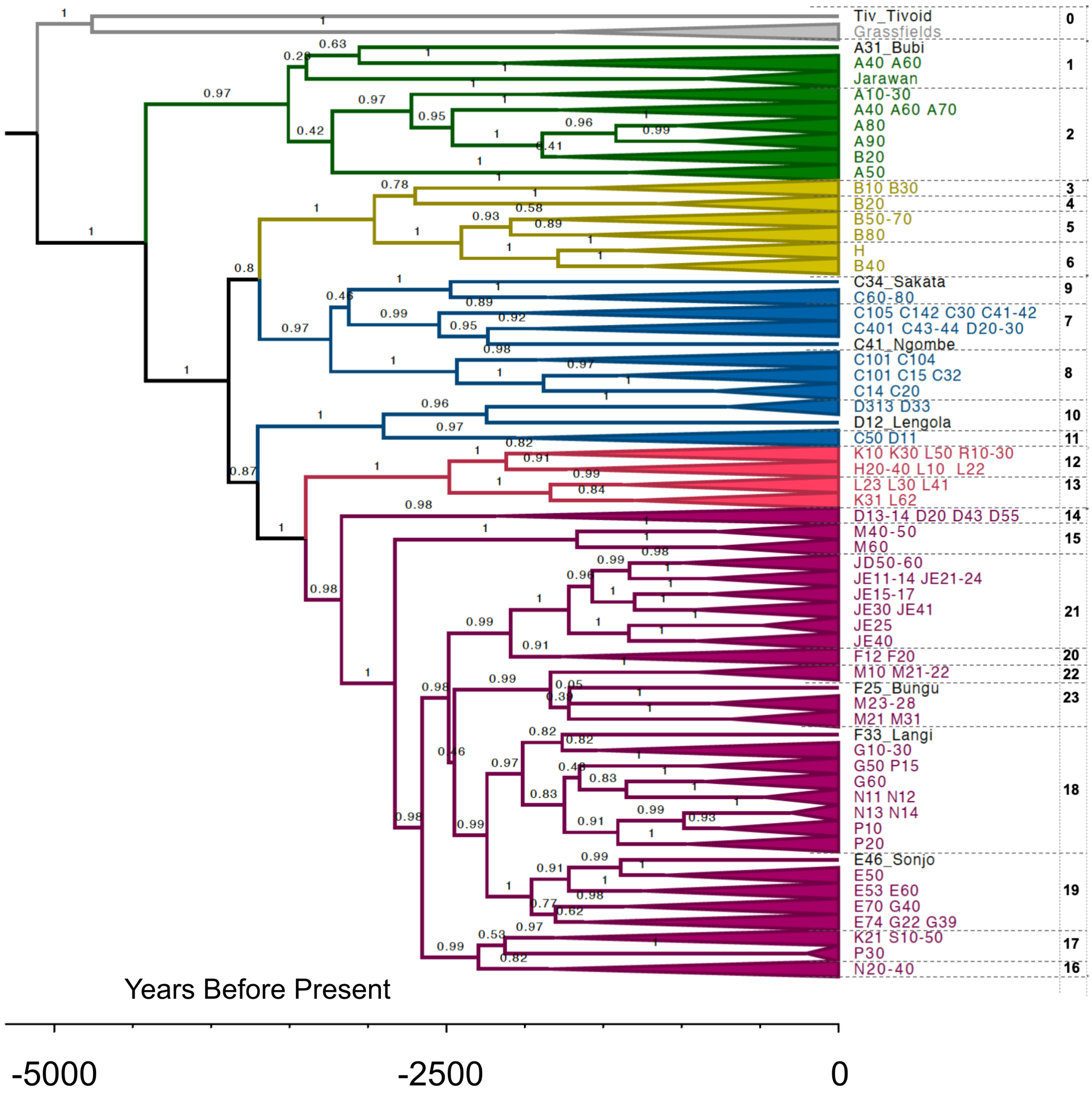

Fig. S3. Phylogeographic tree for the Bantu languages. Detail of languages in each clade. Compare with Figure 2

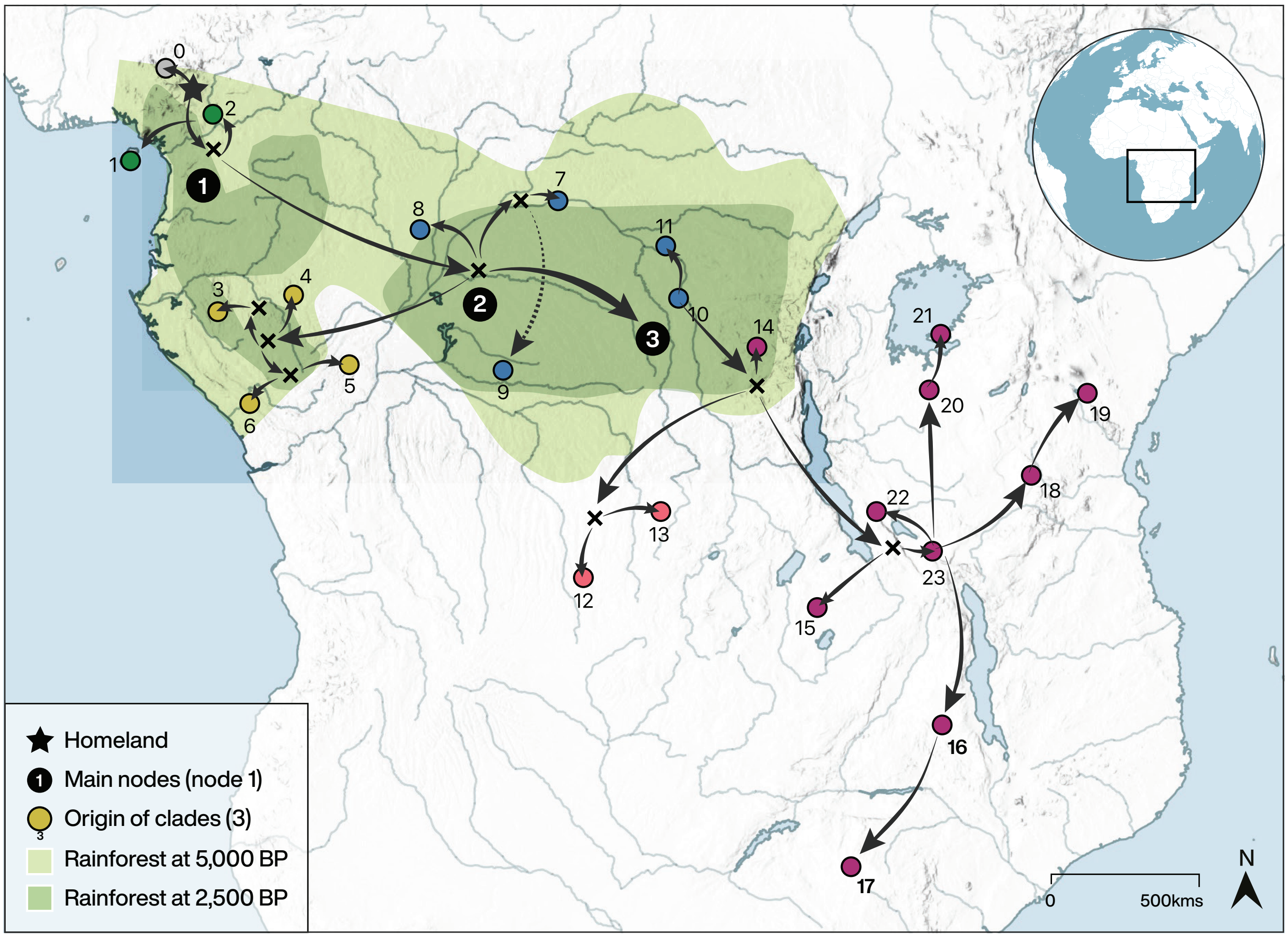

Fig. S4. Bantu migrations reconstructed by using the break-away model in the phylogeographic tree in Figure S3. The homeland is marked with a star, and main nodes are numbered (1-3), as well as main clades (0-23), following the notation and color-coding in Figure 2. Each circle represents the median value of the posterior distribution for the origin of the respective clade (see Figure S5 for greater detail). The span of the rainforest at 5,000 BP and at 2,500 BP is shown, according to (3, 4).

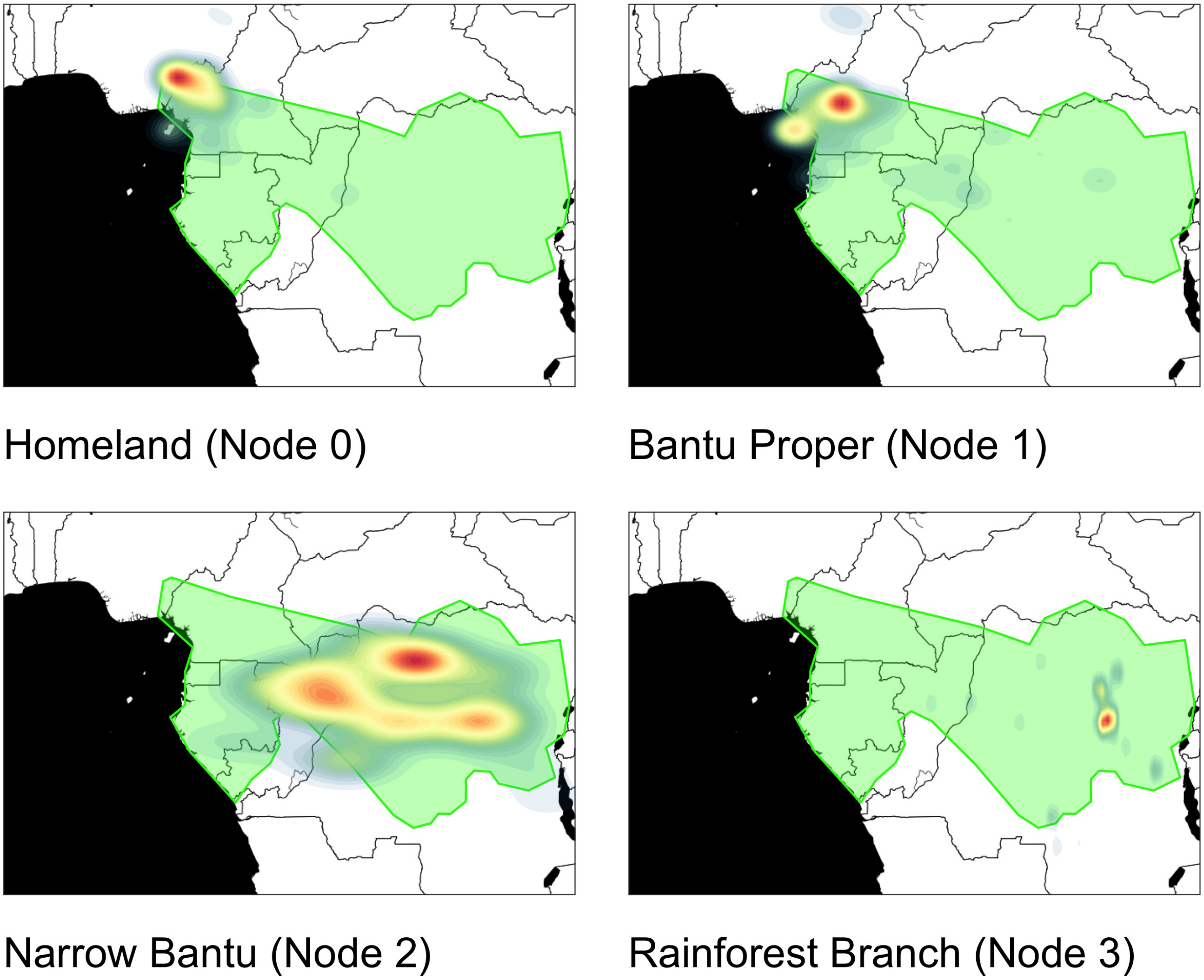

Fig. S5. Heatmaps for the posterior distribution of the locations of the Homeland and Nodes 1-3, as indicated in Figure 2, obtained with the phylogeographic model. These support the fourth hypothesis of a late split and migration through the interior.

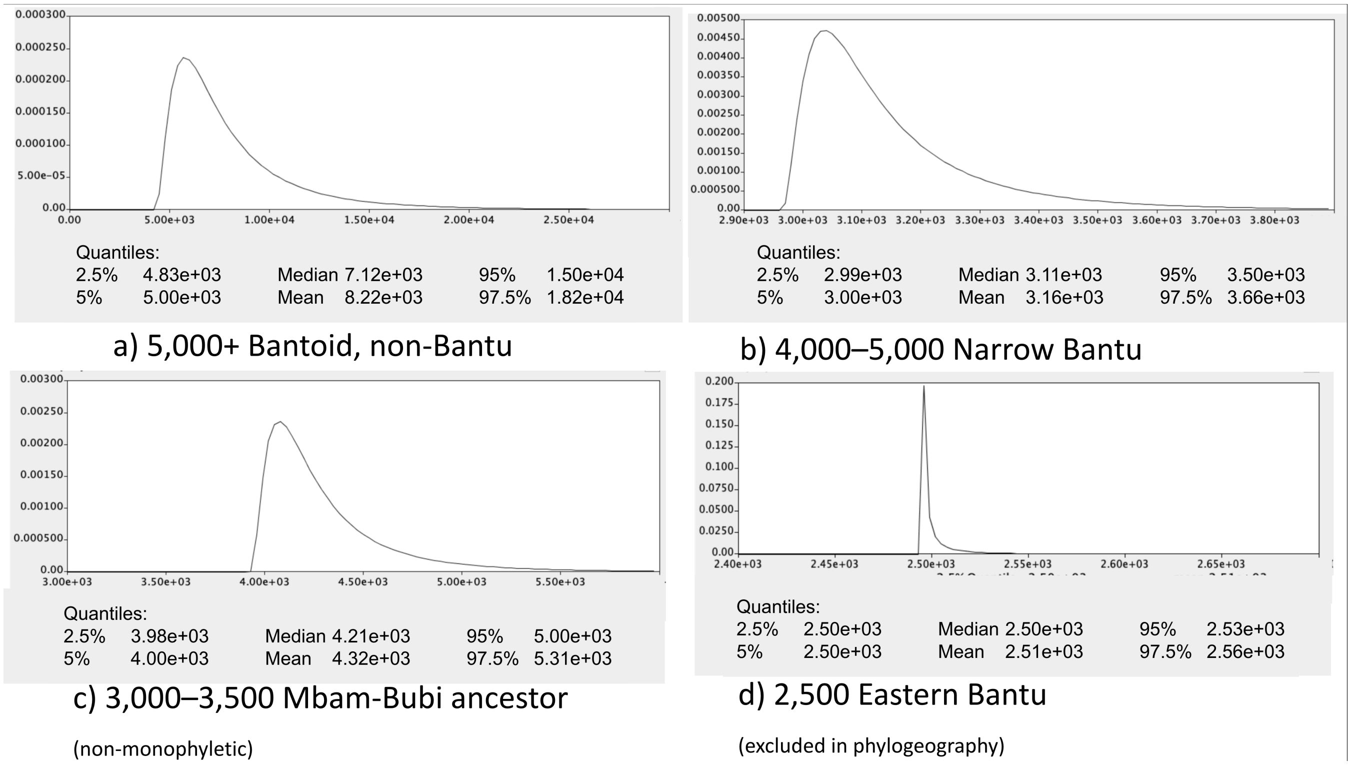

Fig. S6. Calibration points used in our phylogeographic analysis.

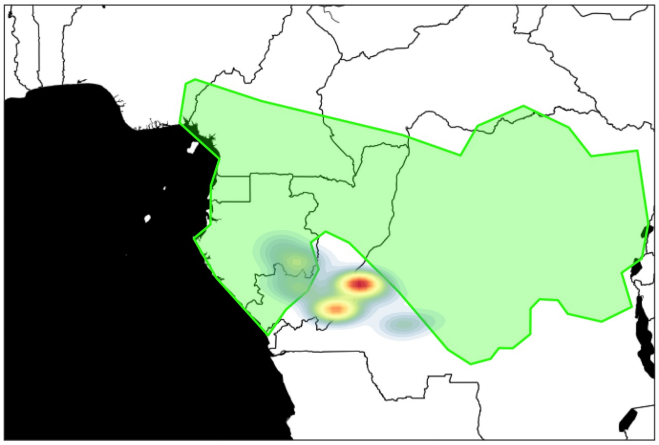

B50-B80 (Clade 5) - Phylogeography

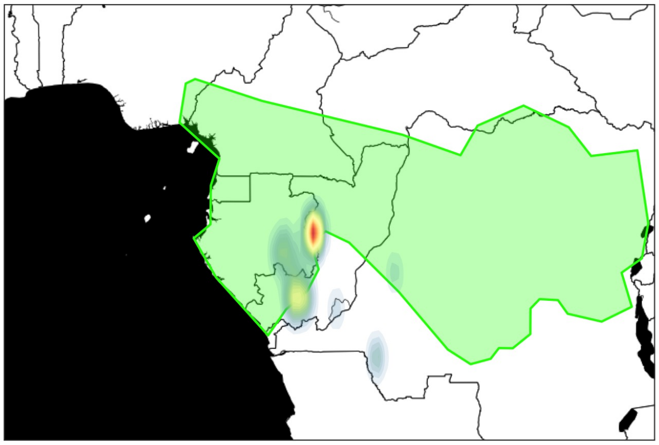

B50-B80 (Clade 5) - Augmented phylogeography

Fig. S7. Heatmaps for the homeland of Coastal Bantu languages (Clade 5 in Figures 2 and S3). We find a location slightly northwestwards from the one found in (5).

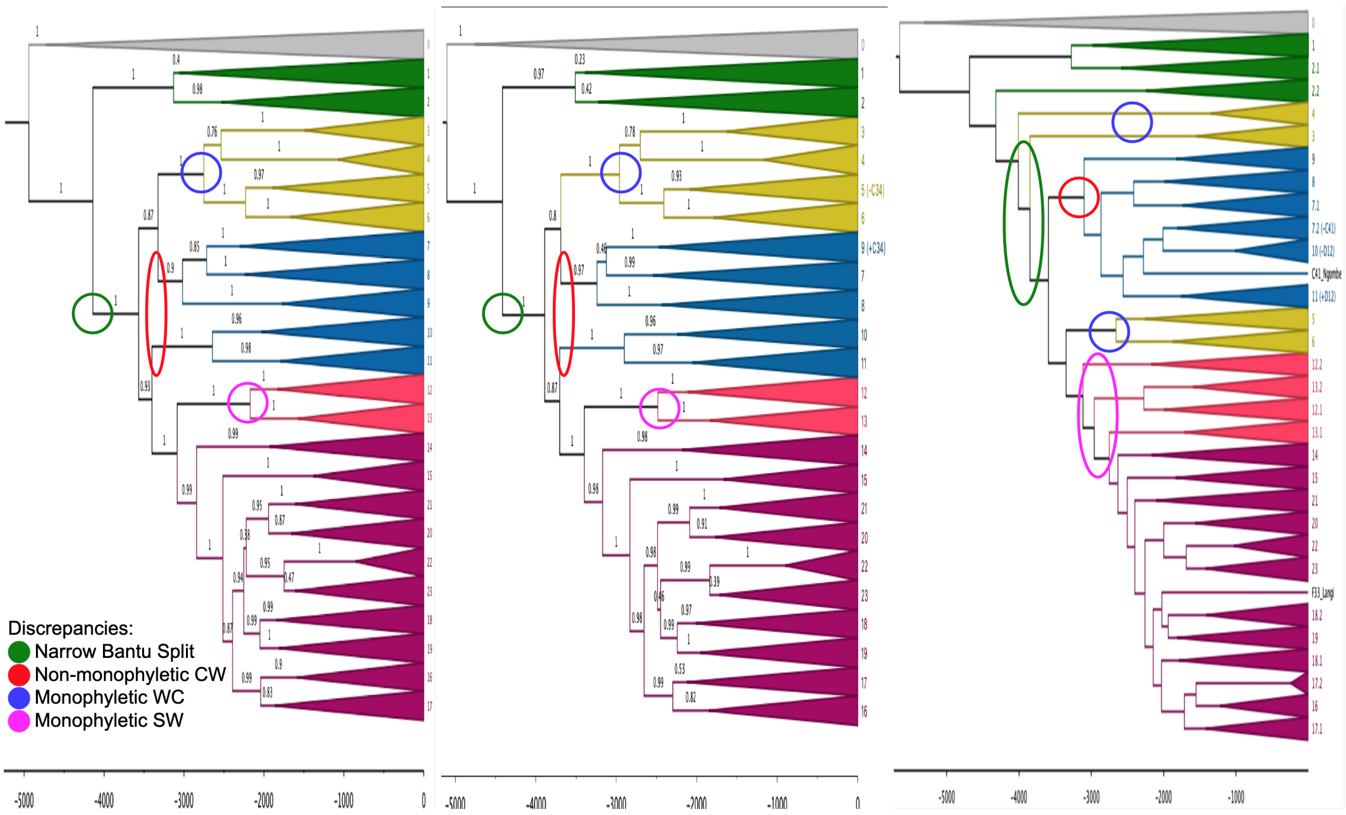

Fig. S8. Comparison of our phylogenetic (left), and phylogeographic (center) analyses with Grollemund et al's 2015 phylogeny (adaptation of their Fig. 1) (right). The main discrepancies are shown as circles and ovals.

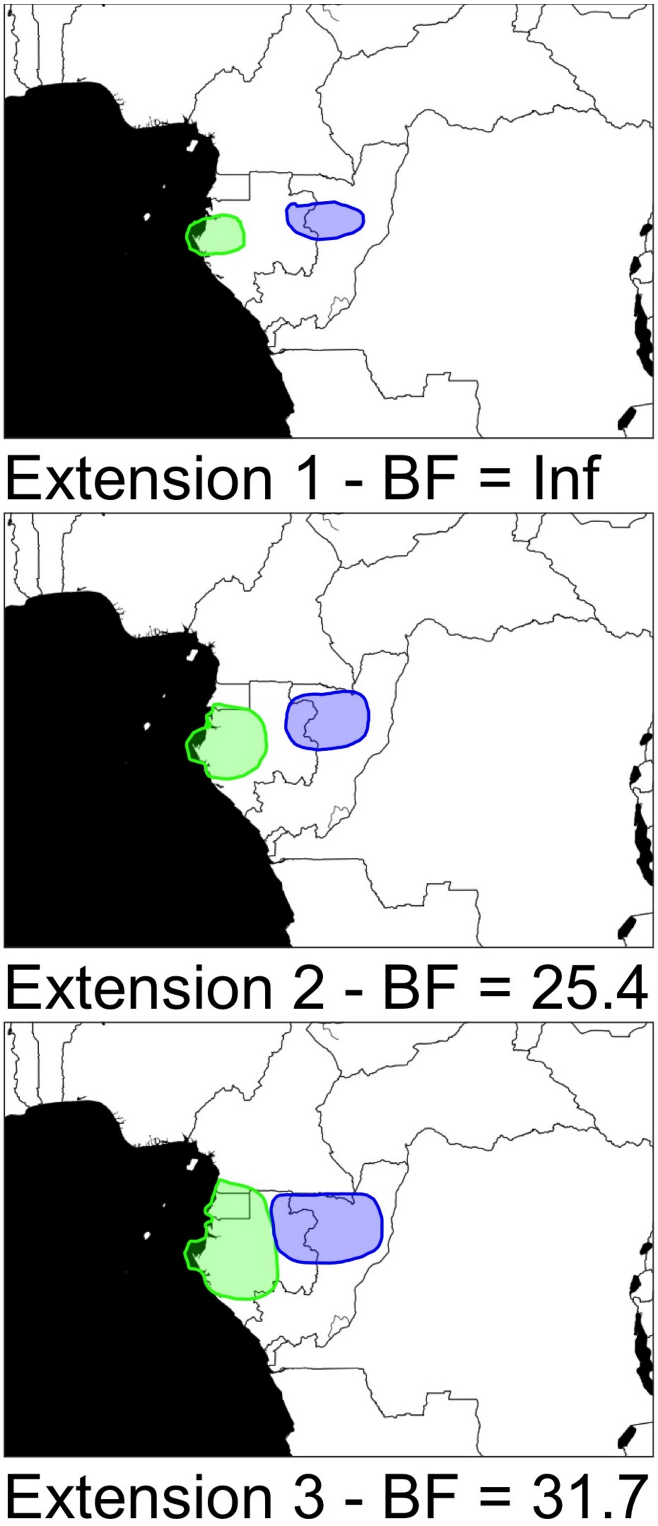

Fig. S9. Areas for the calculation of Bayes factors. In each panel, green area represents the coastal hypothesis, and blue area represents the interior/Sangha hypothesis. Each panel shows a different version of the hypotheses (from the most to the least restrictive).

Table S1. **Model comparison: Results from path sampling**

Name	Site model	Clock	Gamma	Likelihood	Diff. Likelihood
Bantu-alive-ctmc-relaxed-gamma4-stable.25.log	CTMC	relaxed	4	-62910	0.00
Bantu-alive-covarion-relaxed-gamma4-stable.25.log	covarion	relaxed	4	-63042	-132.76
Bantu-alive-ctmc-strict-gamma4-stable.25.log	CTMC	strict	4	-63068	-25.66
Bantu-alive-covarion-strict-gamma4-stable.25.log	covarion	strict	4	-63201	-132.66
Bantu-alive-ctmc-relaxed-gamma1-stable.25.log	CTMC	relaxed	1	-64582	-1381.39
Bantu-alive-covarion-relaxed-gamma1-stable.25.log	covarion	relaxed	1	-64656	-73.54
Bantu-alive-ctmc-strict-gamma1-stable.25.log	CTMC	strict	1	-64835	-179.08
Bantu-alive-covarion-strict-gamma1-stable.25.log	covarion	strict	1	-64915	-80.54

Table S2. Bayes factors calculation

step	coast.post	coast.prior	interior.post	interior.prior	coast.quot	interior.quot	**BF**
1	0.000000	3.105590	7.142857	1.242236	0.0000000	5.750000	**Inf**
2	1.020408	6.832298	15.306122	4.037267	0.1493506	3.791209	**25.38462**
3	1.020408	7.763975	19.387755	4.658385	0.1314286	4.161905	**31.66667**

Data Availability Statement

All code and data are available at https://osf.io/us3q5/?view_only=d54efdad94e3449cae4b533e877b3888
This includes:

SI Dataset S1 (newick_for_export.csv)
List of languages including augmented taxa

SI Dataset S2 (locations_ok.csv)
List of locations

SI Dataset S3 (concept_list.csv)
List of lexical items, from Grollemund et al 2015

SI Dataset S4 (TREES.zip)
xml, log, and tree files for all analyses.

SI Dataset S5 (FINAL_CODE.zip)
Code used for augmenting the tree, generating the heatmaps, and calculating the Bayes Factors.

Figure SS1 Full tree. Linguistic analysis.

Figure SS2 Full tree. Geographic + linguistic analysis.

Figure SS3 Full tree. Augmented Geographic + linguistic analysis.

References

1. Currie, T. E., Meade, A., Guillon, M., & Mace, R. (2013). Cultural phylogeography of the Bantu Languages of sub-Saharan Africa. Proceedings of the Royal Society B: Biological Sciences, 280(1762), 20130695.
2. Grollemund, R., Branford, S., Bostoen, K., Meade, A., Venditti, C., & Pagel, M. (2015). Bantu expansion shows that habitat alters the route and pace of human dispersals. Proceedings of the National Academy of Sciences, 112(43), 13296-13301.
3. Maley, J. (2001). La destruction catastrophique des forêts d'Afrique centrale survenue il ya environ 2500 ans exerce encore une influence majeure sur la répartition actuelle des formations végétales. Systematics and Geography of Plants, 777-796.
4. Maley J (2002) A catastrophic destruction of African forests about 2,500 years ago still exerts a major influence on present vegetation formations. IDS Bull 33(1):13–30.
5. Pacchiarotti, S., Chousou-Polydouri, N., & Bostoen, K. (2019). Untangling the West-Coastal Bantu mess: Identification, geography and phylogeny of the Bantu B50-80 languages. Africana linguistica, 25, 155-229.
